# Stability in skipping gaits

**DOI:** 10.1098/rsos.160602

**Published:** 2016-11-02

**Authors:** Emanuel Andrada, Roy Müller, Reinhard Blickhan

**Affiliations:** 1 Science of Motion, Friedrich Schiller University Jena, Jena, Thüringen, Germany; 2 Institut für Spezielle Zoologie und Evolutionsbiologie mit Phyletischem Museum, Friedrich Schiller University Jena, Jena, Thüringen, Germany

**Keywords:** skipping, locomotion, biomechanics, slip, stability

## Abstract

As an alternative to walking and running, humans are able to skip. However, adult humans avoid it. This fact seems to be related to the higher energetic costs associated with skipping. Still, children, some birds, lemurs and lizards use skipping gaits during daily locomotion. We combined experimental data on humans with numerical simulations to test whether stability and robustness motivate this choice. Parameters for modelling were obtained from 10 male subjects. They locomoted using unilateral skipping along a 12 m runway. We used a bipedal spring loaded inverted pendulum to model and to describe the dynamics of skipping. The subjects displayed higher peak ground reaction forces and leg stiffness in the first landing leg (trailing leg) compared to the second landing leg (leading leg). In numerical simulations, we found that skipping is stable across an amazing speed range from skipping on the spot to fast running speeds. Higher leg stiffness in the trailing leg permits longer strides at same system energy. However, this strategy is at the same time less robust to sudden drop perturbations than skipping with a stiffer leading leg. A slightly higher stiffness in the leading leg is most robust, but might be costlier.

## 1. Introduction

As an alternative to walking and running, humans are able to skip. Unilateral skipping, also known as bipedal galloping, is a temporally and spatially asymmetric locomotion pattern where successive foot falls are not evenly spaced and one leg (leading leg) is always kept in front of the other (trailing leg). Excepting children, this gait is more frequently observed in animals and humans than bilateral skipping (in which both legs periodically work as trailing and leading leg). The reason seems to be that unilateral skipping is easier to coordinate [1,2]. Skipping has a double support phase, as displayed during walking, but it also incorporates flight phases as observed in running. Accordingly, the time course of the centre of mass (CoM) shows features shared by walking (vaulting mechanics) and running (elastic bouncing).

During running and walking, each leg decelerates at first and then accelerates the body; each leg generates a similar amount of braking and propulsive forces. During skipping, the legs act differently. Most of the braking work is executed by the last landing leading leg, while the trailing leg, which lands first after flight phase, provides most of the propulsion [1–4].

Children display skipping gaits at about 4.5 years of age (mainly for behavioural purposes), but cease to use this gait in adulthood (e.g. [1]). This might be related to the higher energetic costs associated with skipping compared with running or walking [5]. At same speeds, skipping consumes about 24% more metabolic energy than running [6]. Still, jerboas, birds like rooks, crows and *Pica pica*, also some lizards, sifakas [7–10] or indrid primates like *Propithecus* [11] use skipping during daily locomotion. Additionally, astronauts from Apollo missions tried many different gaits on the moon's surface and the most preferred was unilateral skipping. Thus, if economy of locomotion does not mainly trigger the use of skipping, one could hypothesize that stability and robustness are the causes of that choice. Recent simulations of a telescopic-legged rimless wheel indicate the existence of stable skipping [12]. The reported stable solutions exist at leg angles and ground reaction force (GRF) profiles that largely differ from those exhibited by humans.

We here test whether the combination of double support phases with aerial phases enable self-stable solutions at speeds at which neither walking nor running display stable solutions. We also ask: (i) about the influence of leg landing angles on skipping dynamics and/or stability, (ii) whether the asymmetric behaviour of the legs during skipping influences global leg parameters such as leg stiffness, and (iii) how stiffness asymmetry influences skipping dynamics and stability. To test our hypotheses, we used a simple bipedal spring loaded inverted pendulum template (BSLIP). By means of this template, skipping was analysed from a global point of view (i.e. through the motion of the CoM and leg function as linear spring). Parameters needed for modelling were obtained from 10 healthy men.

## 2. Material and methods

### 2.1. Experiments

#### 2.1.1. Measurements

Ten male subjects (age: 24.7 ± 2.4 years, body mass: 74.3 ± 6.4 kg, and stature: 180.4 ± 5.7 cm) were instructed to locomote using unilateral skipping along a 12 m runway with one ground level force plate in its centre (9287BA, Kistler, Winterthur, Switzerland). The GRFs were sampled at 2000 Hz. Subjects were allowed to perform several practice trials and were free to choose their trailing leg, namely the first to touch the ground after flight (characterized by the sequence flight–left–right or flight–right–left). After practice, for each subject we recorded six runs slower and six runs faster than the preferred speed. A trial was successful when the subjects skipped across the whole track with touchdown (TD) on the force platform and without losing any reflective joint markers (19 mm). The markers were placed on the tip of the big toe, *lateral malleolus*, *epicondylus lateralis* and *trochanter major* on both sides of the body as well as on *L5* and *C7 proc. spinosus*. All trials were recorded with eight cameras (240 Hz) by a three-dimensional infrared system (MCU 1000, Qualisys, Gothenburg, Sweden) and synchronized by using the trigger of the Kistler soft- and hardwares.

#### 2.1.2. Data processing

Kinetic and kinematic data were analysed using custom written Matlab code (The Mathworks, Inc., Natick, MA, USA). A vertical ground reaction force threshold of 20 N was used to determine the instants of TD and take off (TO). The raw kinematic data were filtered with a third-order low-pass Butterworth filter at 50 Hz cut-off frequency [13]. The distance between *trochanter major* and the middle of the centre of the ball of the foot and *lateral malleolus* was defined as leg length. Leg stiffness was calculated as the ratio between the maximum GRF and the maximum leg compression. As leg length is normally non-symmetric relative to TD and TO events, we averaged leg length during leg contact in order to determine leg stiffness from kinetic data. The results were expressed as mean ± s.d. over all subjects and parameters. To compare kinetic and kinematic parameters, we used repeated measures ANOVAs with *post hoc* analysis (Tamhane tests with Bonferroni correction, SPSS 15.0; SPSS®, Chicago, IL, USA). The homogeneity of variances was assessed using the Levene test.

#### 2.1.3. Simulations

**Figure 1.**
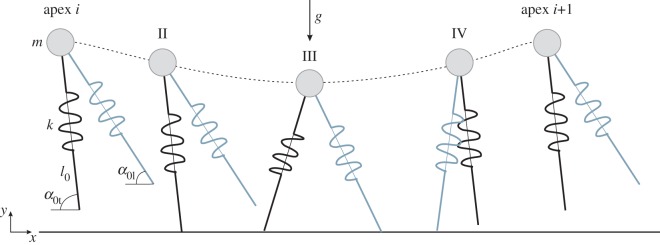
Gait phases during skipping and model parameters. The cycle starts at the highest vertical position (apex). Both legs (black: trailing; grey: leading) are airborne and have different fixed angles with respect to the ground (*α*_0t_, respectively, *α*_0l_). The trailing leg touches the ground first (II). The contact of the leading leg with the ground initiates the double support phase (III). This phase finishes when the trailing leg loses contact (IV). After the leading leg loses contact, the model re-enters the aerial phase. The cycle is completed when the model reaches the apex again. *g*, gravity; *k,* leg stiffness; *l*_0_, leg length at TD; *m*, body mass. Note that for a model having massless legs no difference exists between unilateral and bilateral skipping. The BSLIP model for skipping was first introduced by Minetti [1].

To model skipping with the BSLIP (which is similar to Minetti's model [1]), we need at least one more parameter than normally used to model walking or grounded running. This is because each leg has a different angle of attack when contacting the ground (figure 1). The number of parameters can further increase by considering, for example, different stiffness or different leg length values for each leg. For the sake of reducing parameter space, we assumed the same value in both legs for leg stiffness and leg length at TD. In experiments, leg stiffness and leg length at TD were not significantly different between legs (table 1). Thus, the model presented here has six parameters (gravity *g,* leg stiffness *k*, angle of attack of the trailing leg *α*_t_, angle of attack of the leading leg *α*_l_, body mass *m*, leg length at TD *l*_0_). However, as skipping entails aerial phases, stability considerations can be reduced to a one-dimensional *apex* return-map as shown for running [14]. Therefore, we defined such subspace for our system using the *apex* of the CoM trajectory (*y* = *y*_max_ and $\dot{y}=0$; i.e. inversion from positive to negative). Then for a given speed, the CoM state at the *apex* at step *i* is defined by *y_i_*. We have neglected two- or more-periodic solutions. Fixed points in this map represent periodic solutions of the continuous time system and fulfil *y_i_*_+1_(*y_i_*) = *y_i_*.

**Table 1.** Kinetic and kinematic parameters separated for trailing and leading leg. Mean ± s.d. across all trials for investigated kinetic and kinematic parameters: maximal vertical GRF; leg length and leg angle at TD; leg stiffness. Significant differences (*p* < 0.05) between slow and fast are marked in italic. *N* denotes number of subjects; *n*, number of total trials.

	slow	fast
trailing leg		
skipping speed	2.7 ± 0.2^b^	*3.7 ± 0.5*^b^
GRF (N)	1601 ± 211^b^	*1792 ± 166*^b^
leg length at TD^a^	1.05 ± 0.01	1.04 ± 0.01
leg angle at TD (degree)	75.2 ± 2.2^b^	*78.5 ± 3.7*^b^
leg stiffness (N m^−1^)	21 783 ± 4785	*29 748 ± 5895*
*N* (*n*)	6 (33)	6 (35)
leading leg		
skipping speed	2.4 ± 0.2^b^	*3.4 ± 0.2*^b^
GRF (N)	1418 ± 144^b^	1505 ± 77^b^
leg length at TD^a^	1,04 ± 0.01	1.04 ± 0.01
leg angle at TD (degree)	52.0 ± 2.5^b^	53.2 ± 1.9^b^
leg stiffness (N m^−1^)	20 584 ± 4398	*29 693 ± 8687*
*N* (*n*)	4 (23)	4 (22)

^a^Relative value (leg length at ground contact/leg length in standing position).

^b^Significant differences between trailing and leading leg.

Based on our experimental data and our hypotheses, we investigated the parameter space ranging from 820 J ≤ *E* ≤ 1500 J, 3 kN m^−1^ ≤ *k* ≤ 40 kN m^−1^, 48° ≤ *α*_l0_ ≤ 90, 68° ≤ *α*_t0_ ≤ 105° and 1.01 m ≤ *y_i_* ≤ 1.2 m for periodic solutions. Note that our parameter search is broader than the values of *k* and *α* so far obtained for bird or human walking, grounded running or running [15,16]. Applying a Newton–Raphson algorithm in this iteration [17], we identified a fixed point when deviations |*y_i_*_+1 _− *y_i_*| were less than 10^−9^. Moreover, as a sufficient condition, the slope d*y_i_*_+1_(*y_i_*)/d*y_i_* within a range of (−1, 1) in the neighbourhood of the fixed point indicates the stability of the movement pattern. This means that after a perturbation, the system returns asymptotically to the same periodic oscillation without any feedback control.

For some representative stable solutions, we induced an asymmetry in the values of the leg stiffness *k* to infer their influence in the response of the system*.* For the sake of simplicity, we just increased or decreased the stiffness in the trailing leg starting while maintaining system energy *E*. We then applied a rather intuitive method (step-down step-up) to identify the robustness of the selected solutions. After such a sudden drop perturbation, the energy of the system is the same as prior to it. SLIP models are not able to abolish perturbation with respect to energy. The same sudden drop perturbation was used to check the robustness of some solutions with more retracted legs at TD (*α*_t0_ > 90°). To integrate the equations of motions, we applied a variable-step algorithm (ode45) with a relative and absolute integrator error tolerance of 1 × 10^−9^ and 1 × 10^−10^.

## 3. Results

### 3.1. Experiments

Six subjects contacted the force plate with the trailing leg and four subjects contacted the force plate with the leading leg. Significant differences between slow and fast skipping were more obvious in the trailing leg than in the leading leg (table 1). More precisely, in the trailing leg vertical GRFs, leg stiffness and leg angle at TD increased with skipping speed. In the leading leg, only leg stiffness increased with speed (table 1). Independent of skipping speed, as compared to the leading leg, in the trailing leg we found significantly higher GRFs and leg angles at TD. As compared to the leading leg, leg stiffness in the trailing leg was measured to be slightly higher but not significant (table 1).

### 3.2. Simulations

**Figure 2.**
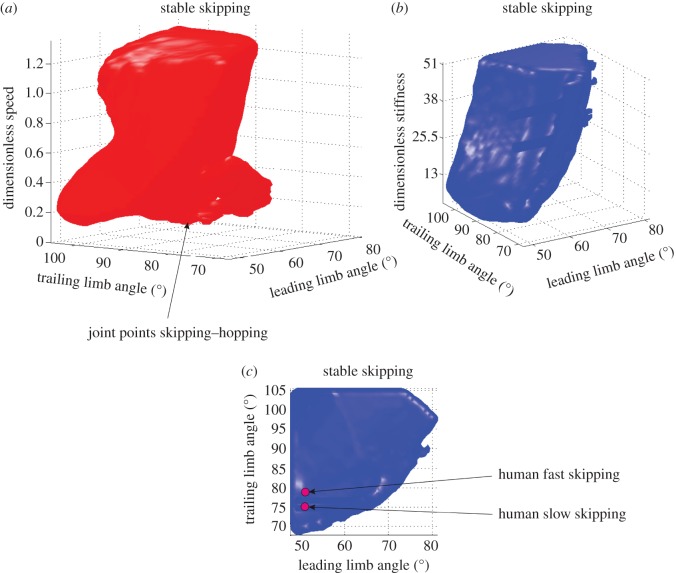
Fields of stable skipping gaits. To facilitate comparison between species, we present data dimensionless. (*a*) Leg angles at TD versus dimensionless speed ($\hat{u}=v\text{/}\sqrt{gl_{0}}$). (*b*) Leg angles at TD versus dimensionless stiffness ($\hat{k}=kl_{0}\text{/}mg$). Leading refers to the leg that is foremost. Trailing leg touches the ground first.

**Figure 3.**
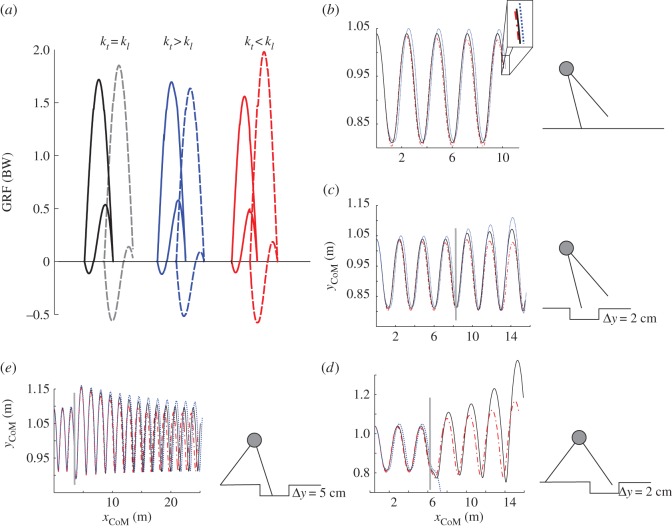
Influence of leg stiffness asymmetries on (*a*) profile of GRF, (*b*) distance travelled and (*c–e*) robustness against sudden drops. (*a*) Solid line, trailing leg; dashed line, leading leg. Left, both legs have same stiffness (black), middle, trailing leg 5% higher stiffness (blue); right trailing leg 5% lower stiffness (red). (*b–e*) Both legs have same stiffness (black), trailing leg 5% higher stiffness (dot blue); trailing leg 5% lower stiffness (dashed-dotted red). (*b*) Larger distances can be achieved with higher stiffness in the trailing leg. (*c,d*) For skipping with relatively more protracted leg, as observed in humans, higher stiffness in the leading leg is more robust against perturbations. (*e*) Skipping gaits that use a more retracted trailing and leading legs are more robust to sudden perturbations. Simulation parameters for (*a–d*): *α*_t0_ = 75°; *α*_l0_ = 55°; *E* = 1440 J; *y*_0_ = 1.0389 m; *k* = 12 000 N m^−1^. Simulation parameters for (*e*) *α*_t0_ = 91°; *α*_l0_ = 71°; *E* = 1300 J; *y*_0_ = 1.0934 m; *k* = 38 000 N m^−1.^

We found large fields of stability (figure 2). Stable solutions exist for all the mapped values of *k*, *E* and *α*_t_. For *α*_l_, we found stable solutions at *α*_l_ between 48° and 84°. Those stable solutions of the BSLIP capture important features of skipping kinetics. As observed in the experimental data, the trailing leg mostly accelerates, and the leading leg mostly brakes (figure 3*a*).

#### 3.2.1. Influence of speed

The leading leg angle at TD has little influence on stability independent of speed. The contrary is true for the trailing leg. For example, for trailing leg angles higher than 95° skipping is stable across almost the whole speed range. For a more protracted trailing leg at TD (smaller angles), a stable solution exists at higher speeds. Thus, if the trailing leg contacts the ground with an angle of attack of 70°, skipping is only stable at speeds higher than $\hat{u}=1.2$.

Interestingly, stable skipping exists from very low speeds (e.g. $\hat{u}=0.0014$; ≈ 0 m s^−1^ for a human) up to running speeds ($\hat{u}=1.35$ at the highest energy we mapped; *ca* 4.2 m s^−1^ for a human). In general, across most of this speed range skipping is stable at all the leg stiffness values that we mapped. For speeds higher than $\hat{u}=1.0$ and leg stiffness $\hat{k}>5$, stable solutions can be observed. For speeds below $\hat{u}=0.2$, stable solutions exist for leading leg angles below 80°, and trailing leg angles above 95°, but for the range of leg stiffness we tested. The lowest speeds with stable skipping are observed for close to symmetric leg angle configurations and for higher leg stiffness ($\hat{k}>30$). Here, skipping merges with hopping (e.g. 78° for the leading leg and 102° for trailing leg).

#### 3.2.2. Influence of leg stiffness

Asymmetry in leg stiffness has considerable influence on the locomotor efficiency (system energy/distance travelled) and robustness against sudden drops.

When trailing and leading legs have same leg stiffness *k*, the peak value of the vertical component of the GRF exerted by the trailing leg is always lower than that of the leading leg. The gap between those peaks varies for different leg configurations.

Variations up to ±6% in the stiffness of the trailing leg still produce stable steady-state solutions for the same parameters that produced stable skipping with same *k* in both legs. Higher stiffness in the trailing leg increases peak GRF in the trailing leg and reduces that of the leading leg. The contrary is true for higher stiffness in the leading leg. Anterior posterior forces increase and decrease in proportion (figure 3*a*). For the example displayed in figure 3*a*, 5% higher stiffness in the trailing leg exerted 3% higher vertical GRF in this leg and a reduction of 10% in the vertical GRF of the leading leg. 5% less stiffness in the trailing leg produced a decrement of 5% and an increment of 12% in the peak value of the vertical GRF of the trailing, respectively leading leg. Additionally, higher stiffness in the trailing leg with respect to the leading leg permits travelling longer distances with the same energy (figure 3*b*). The higher this asymmetry, the larger the travelled distances. The contrary is true for higher stiffness in the leading leg. However, a stiffer leading leg makes skipping more robust to sudden drop perturbations when the trailing leg drops inside the step-down perturbation (figure 3*c*). In the case presented in figure 3*b*, 5% higher stiffness in the trailing leg resulted in a travelled distance, which was 1.6% longer than that achieved with legs having same stiffness and 2.6% longer than the distance reached with 5% lower stiffness in the trailing leg at the same energy level.

#### 3.2.3. Influence of leg angles at touchdown and of the leg stepping into the perturbation

Larger trailing leg angles (leg more retracted) allow broader combination possibilities with leading leg angles that ensure stability (figure 2). In addition, these leg configurations are more robust against perturbations but they may be energetically less beneficial.

**Figure 4.**
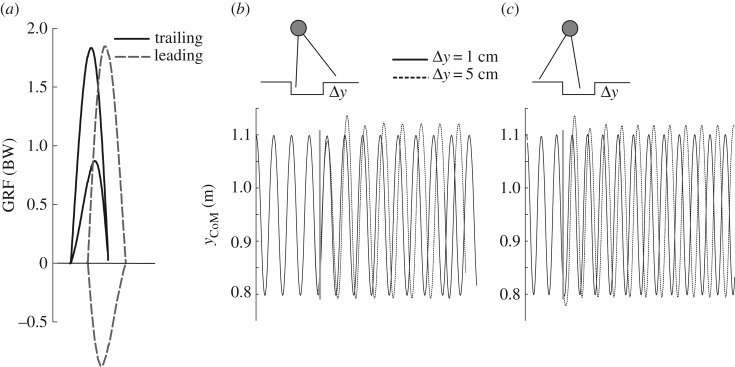
Skipping with a retracted trailing leg (leg angle > 90°). (*a*) Even terrain. The trailing leg only accelerates, and the leading leg in most of the cases only decelerates. (*b,c*) The use of a more retracted trailing leg is more robust against larger perturbations. (*b*) Trailing leg drop-in perturbation. (*c*) Leading leg drop-in perturbation. In (*b,c*), the oscillations of the CoM from two simulations with a step-down step-up perturbation are shown (1 cm, respectively, 5 cm). Note that after the 1 cm perturbation, the model returns to the same fixed point (periodic oscillation). The model can also deal with a 5 cm perturbation; however in this case, the model shifts to a different fixed point. Obviously, for this parameter combination at least two stable limit cycles exist. Simulation parameters: *α*_t0_ = 101°; *α*_l0_ = 57°; *E* = 940 J; *y*_0_ = 1.0998 m; both legs have same *k* = 19 000 N m^−1^.

When the trailing leg is retracted to angles larger than 90°, the trailing leg accelerates only and the leading leg in most cases only brakes (figure 4*a*). This strategy is very resistant to sudden drops independent of which leg steps into the perturbation. Indeed, in all trials we perturbed, we could observe a very robust fixed point or even a second stable limit cycle for the same model parameters for larger perturbations (normally for trailing leg angles larger than 92°; figure 4*b*).

When the leading leg drops into the perturbation with a more protracted leg angle (values similar to those observed in human skipping), the BSLIP response becomes less stable (figure 3*d*). This behaviour changes to a very robust one if the legs are held more retracted during the swing phase (figure 3*e*). However, locomoting with more retracted leg necessitates more steps to cover the same distance.

## 4. Discussion

While spring–mass running is stable at locomotion speeds higher than 3 m s^−1^ ∼ $\hat{u}=0.96$ [18] and walking only within a narrow corridor around 1 m s^−1^ ∼ $\hat{u}=0.32$ [19], skipping is stable across an amazing speed range. Skipping covers speeds from hopping to running and fills the complete energy gap between spring–mass walking and running. We have previously hypothesized that this gap would be filled up by spring–mass grounded running, but our results revealed that this gait is unstable without a pronograde trunk [15,20].

Our model predicted peak vertical GRFs for the leading leg that are higher than those exerted by the trailing leg. This result was surprising at first. Kinetic data collected during level skipping display that the peak of the vertical GRF exerted by the trailing leg is normally slightly higher than the peak observed for the leading leg (in our case significant; table 1). In terms of our model, this would mean that the stiffness in the trailing leg is higher (figure 3*a*). This prediction is in accordance with our experimental findings (table 1). We estimated the stiffness in the trailing leg to be slightly but not significantly higher.

In our simulations, higher leg stiffness in the trailing leg turns out to be appropriate for level locomotion as it is more efficient (with the same energy the model travels long distances; see figure 3*a,b*). This finding might explain the use of the higher stiffness in the trailing leg. However, this strategy is at the same time less robust to sudden drop perturbations. For this case, slightly higher stiffness in the leading leg becomes the best option (figure 3*c,d*), but at the cost of larger asymmetries in leg forces, and hence less economic locomotion, as shown for human walking [21] and avian mixed gaits [22]. For the leg configurations, we observed during human skipping (relative protracted) that the leading leg is somewhat less robust to sudden drop perturbations (figure 3*d*). For those leg configurations, better performances may be achieved by the use of a retraction or lengthening policy in the leading leg [23,24]. These topics must be addressed in future experimental and simulation studies.

Skipping with a trailing leg retracted to angles larger than 90° turns out to be very robust against sudden drops despite which leg contacts in the perturbation. Interestingly, for trailing leg angles larger than 92° two stable fixed points exist for the same model parameters (figure 4*b,c*). Both solutions are skipping gaits but with different oscillation amplitudes. The flipside of this strategy is that it may expend more energy. It needs more steps to cover the same distance than skipping with more retracted legs. Still, due to its robustness, this strategy might be adequate for facultative bipeds such as lizards or non-human primates. However, further analyses aimed more directly at assessing energy costs related to leg stiffness are necessary to support the results presented here.

As observed for mixed gaits [22], the use of skipping again indicates the existence of a trade-off between stability and economy of locomotion. Skipping consumes 24–30% more metabolic energy and 15% more mechanical energy than running at the same speed [5,6,25]. Collision models predicted that the double contact and the velocity inversion might be the causes of higher cost of locomotion [26]. This prediction was not supported by experimental findings recently published, as no redirection of the CoM occurred during double support [6]. Likelier, higher cost is related to higher power generation in the hip during skipping. The proximal muscles such as the hip extensors in humans do not have long tendons, as found in the distal muscles [27], which makes power generation costlier compared with power generation at the ankle. However, the influence of hip power might be less important for crouched leg configurations that may explain the use of skipping in small birds such as crows. Small birds tend to minimize the rotation of the femur and optimize the use of elastic recoil in the distal joints for economy in locomotion [28]. The moments and power data that Fiers and colleagues presented for the ankle and knee during unilateral skipping indicate an energy-saving mechanism [6]. They also found that positive work at the ankle and knee is preceded by negative work during the stance phase. Thus, they concluded that there is potential for elastic energy storage and recoil in unilateral skipping as already anticipated in [1].

The leg behaviour explained above permits the use of the BSLIP for modelling purposes, although it represents a strong mechanical abstraction of the system. We assumed a linear leg stiffness estimated from the force–length function of the leg that represents the global action of leg muscles and tendons. This process is also not free from flaws. In human skipping, the leg length differs with respect to TD and TO events. The BSLIP is not able to display such leg asymmetries when simulating skipping. Therefore, we averaged leg lengths during stance in order to determine leg stiffness from kinetic data. Furthermore, we addressed only small asymmetries in the leg stiffness (around 6%) in our modelling studies that are of the order of the observed differences. Future studies need to address systematically the influence of higher leg stiffness asymmetries as well as the influence of trunk orientation on skipping.

As skipping combines double support phases with aerial phases, system energy can be managed between the legs to produce different system behaviours. Accordingly, we found plenty of combinations of leg angles and leg stiffness that produce stable skipping. In addition, skipping is very robust against errors by setting parameters, and it can be used within a large speed range. In nature, locomotion stability might be preferred in some situations over economy of locomotion, and therefore skipping gaits adopted. In reduced gravity, experiments and models indicate that skipping might be energetically beneficial [29,30]. In addition, Pavei *et al.* [30] suggested that balance (stability) could have been a mechanical trigger for the choice of skipping on the moon. This assumption is now supported by our simulations. In summary, skipping is a stable and robust gait, a great exercise and can bring a lot of fun not only to children.

## Supplementary Material

kine

## Supplementary Material

force

## Supplementary Material

FixSkipping_All

## Data accessibility

Kinematic and kinetic data as well as simulation results are in the electronic supplementary material. Simulation results include stable and unstable fixed points.
